# High Prevalence of HIV-1 Intersubtype B′/C Recombinants among Injecting Drug Users in Dehong, China

**DOI:** 10.1371/journal.pone.0065337

**Published:** 2013-05-31

**Authors:** Xiaoxu Han, Minghui An, Bin Zhao, Song Duan, Shaomin Yang, Junjie Xu, Min Zhang, Jennifer M. McGoogan, Yutaka Takebe, Hong Shang

**Affiliations:** 1 Key Laboratory of AIDS Immunology of Ministry of Health, Department of Laboratory Medicine, The First Affiliated Hospital of China Medical University, Shenyang, Liaoning Province, China; 2 Dehong Prefecture Center for Disease Control and Prevention, Mangshi, Yunnan Province, China; 3 AIDS Care Center, Yunnan Provincial Hospital Infectious Disease, Kunming, Yunnan, China; 4 National Center for AIDS/STD Control and Prevention, Chinese Center for Disease Control and Prevention, Beijing, China; 5 AIDS Research Center, National Institute of Infectious Diseases, Tokyo, Japan; Institut Pasteur of Shanghai, Chinese Academy of Sciences, China

## Abstract

**Objective:**

To examine the distribution of HIV-1 genotypes among injecting drug users (IDUs) from Dehong, Yunnan province.

**Materials and Methods:**

Blood samples from a total of 95 HIV-positive IDUs were retrospectively analyzed. Samples were collected between 2005 and 2009 from four cities in Dehong prefecture, western Yunnan province, the geographical origin of the HIV epidemic in China. HIV-1 *gag*, partial *pol*, *vpr-env* fragment, half-genome, or near-full-length sequences were analyzed to determine the HIV-1 genotypes of each subject. Results were compared with findings from past studies of IDUs in Dehong and in neighboring Myanmar.

**Results:**

We observed a high prevalence of B′/C recombinants (82.4%) among IDUs in Dehong, the structural profiles of which do not match those previously reported in Dehong or in Myanmar. Furthermore, statistically significant differences in geographical and temporal distributions of HIV-1 genotypes were characterized by a predominance of HIV-1 B′/C recombinant forms among older subjects(p = 0.034), subjects from Longchuan district (p = 0.022), and subjects diagnosed between 2000 and 2004 (p = 0.004).

**Conclusions:**

The increasing prevalence of multiple, new B′/C recombinant forms suggest that HIV-1 intersubtype recombination is substantial and ongoing in western Yunnan. This reflects the high-risk behavior of IDUs in this region and argues the need for stronger monitoring and prevention measures in Dehong and other high-prevalence areas around China.

## Introduction

**Figure 1:**
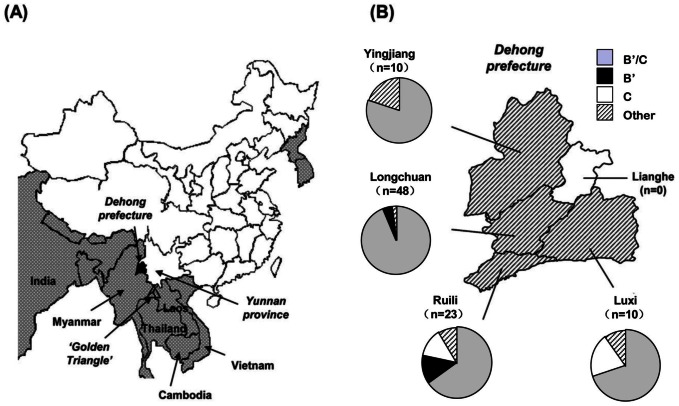
Maps depicting study sites and the geographical distribution of HIV-1 genotypes. (A) Illustration of the geographical location of Dehong prefecture (Dehong Dai-Jingpo autonomous region) in Yunnan Province, southwestern China, with the “golden triangle” (▵) region highlighted as a nearby area of significant opiate drug trafficking. (B) Illustration of the geographical location of the four districts within Dehong prefecture observed in this study and the numbers of subjects from each district. Pie charts depict HIV-1 genotype distribution in each of these districts. The category labeled “other” includes CRF01_AE, C/CRF01_AE and B′/C/CRF01_AE genotypes.

Yunnan province, located in southwestern China, was the first province to report evidence of an HIV epidemic and remains the most severely affected area in China today. The Dehong, Dai-Jingpo autonomous prefecture is one of eight minority autonomous prefectures in Yunnan province. According to China's 2010 national census, Dehong prefecture had a total population of approximately 1.2 million people with 49% belonging to minority groups, including Dai, Jingpo, Achang, Lisu, and Deang. Dehong encompasses an area of 11,526 square kilometers, divided into five districts: Luxi, Ruili, Longchuan, Yingjiang, and Lianghe (Figure 1A). Except for Lianghe, each district shares a border with Myanmar. Travel, commerce, and intermarriage across the China-Myanmar border are common in this region.

Dehong has a long history of opiate trade, and the vast majority of illicit drugs in China are trafficked through Dehong from the “golden triangle,” which is comprised of large regions of Myanmar, Thailand, Laos, and Vietnam (Figure 1A). In 1989, HIV-1 was first detected in injecting drug users (IDUs) in Ruili [1]–[4], and shortly thereafter in Longchuan and Luxi, all within Dehong prefecture [5]–[7]. These events marked the beginning of a true HIV epidemic in China. In 1992, a study examining HIV among IDUs in Dehong revealed that the prevalence of HIV infection had reached 81.8% in Ruili and 44.6% in Longchuan [6]. The spread of HIV in China was rapid and by 1995, outbreaks had been detected among IDUs in Sichuan, Xinjiang, and Guangxi provinces [8], [9].

Over the past two decades, both field studies and molecular epidemiology surveys have identified Yunnan province, and specifically Dehong prefecture, as the epicenter of the HIV/AIDS epidemic in China. HIV-1 strains circulating in Dehong have shown extremely high genetic diversity [8]–[11]. While the HIV-1 epidemic among IDUs in Yunnan was originated by both HIV-1 subtype B and subtype B′ (Thailand variant of subtype B) strains [11]–[16], subtype B′ had become the dominant strain among IDUs by the early1990s [11]–[17]. HIV-1subtype C strains were first identified among IDUs in Ruili and Longchuan in 1992 [11]–[16], [18], and subsequently two circulating recombinant forms (CRFs), CRF07_BC and CRF08_BC, several B′/C unique recombinant forms (URFs), and CRF01_AE were documented among IDUs in Dehong [11]–[16]. By 1997, CRF07_BC and CRF08_BC had begun to spread widely among IDUs in China [8]–[11]. It has been speculated that CRF07_BC and CRF08_BC arose in Yunnan province, most likely in Dehong prefecture, and spread via two different overland heroin trafficking routes: CRF07_BC toward the north to Xinjiang and Liaoning provinces [8]–[11], and CRF08_BC toward the east to Guangxi province [8], [10]. However, another study has demonstrated that CRF07_BC spread to other regions of mainland China from Xinjiang. Thus, while the C and B′ fragments in *gag-pol* region are known to be from Yunnan [19], the geographic origin of CRF07_BC is still yet to be definitively determined.

As the most recent molecular epidemiology study of HIV-1 in Dehong was performed before 2005 [11], [15], [16], the current status and trends of the HIV epidemic at the molecular level among IDUs in this region is largely unknown. Adjacent Myanmar is considered a geographical “hot-spot” of extensive HIV-1 subtype recombination with some 86.1% of HIV-positive IDUs in northern Myanmar carrying B′, C, and CRF01_AE recombinant strains of the virus. Although Dehong is expected to be in a similar situation because of the extensive trade and travel over the China-Myanmar boarder, the relationship between the HIV epidemics in Dehong and Myanmar remains unclear [20].

The primary aim of this study was to examine the distribution of HIV-1 genotypes among IDUs in Dehong, with secondary aims of investigating potential associations with demographic and epidemiological features of the study population and examining the current distribution in comparison to past findings.

## Methods

### Study population and blood sample collection

A total of 95 individuals were included in this study. Subjects were selected based on the following criteria: 1) self-reported acquisition of HIV through injecting drug use, 2) residence in one of the four Dehong districts that border Myanmar, and 3) enrollment between 2005 and 2009, at either the HIV/AIDS Care Center of Yunnan province or the Dehong Center for Disease Control and Prevention (Dehong CDC). Existing demographic information and clinical data as well as EDTA-3K-treated, anti-coagulated whole blood samples were acquired for each subject. Samples were centrifuged at 300 g for 10 minutes and plasma was aliquoted and stored at −80°C.

### RNA extraction, amplification, and sequencing of HIV-1 gene fragments

RNA was extracted from 280 µl of plasma using QIAamp® Viral RNA Mini Kit (Qiagen, Germany) in a final elution volume of 60 µl. The sequences within the *gag* gene (HXB_2_ 790–2292), partial *pol* gene (HXB_2_ 2253–3318), and *vpr-env* region (HXB_2_ 5671–6449) fragments were amplified according to methods described in the literature [21]–[23]. In brief, HIV-1 *gag*, partial *pol* and *vpr-env* gene fragments were reverse-transcribed and amplified with SuperScript™ Polymerase One-Step RT-PCR System (Invitrogen, USA) and specific outer primers, respectively. Second round PCR amplification was performed using GoTaq DNA Polymerase (Promega, USA) and specific inner primers. PCR products were purified using QIAquick Gel Extraction Kit (Qiagen, Germany) and sequenced directly as described previously [21]–[23].

### Single genome amplification and sequencing of HIV-1near-full-length genomes

The 5-kb 5′and3′ half-genomes were amplified from RNA in plasma via RT-PCR with SuperScript™ III Reverse Transcriptase and Platinum Taq DNA Polymerase High Fidelity (Invitrogen, USA) as previously described [24]. Single genome amplification (SGA) and sequencing of HIV-1 DNA were performed in order to acquire the dominant single virus sequence from quasi-species [24]. Amplicons were sequenced directly by Beijing Genomics Institute (China) using internal walking primers.

### Phylogenetic tree and recombination breakpoint analyses

Using the Los Alamos HIV Sequence Database (http://www.hiv.lanl.gov), all sequences were screened by the HIV BLAST tool to confirm lack of laboratory contamination and then aligned with HIV-1 reference strains. Alignment and manual editing were accomplished using Clustal X software (Version 2.0) and BioEdit software (Version 7.0; http://www.mbio.ncsu.edu/bioedit/bioedit.html), respectively. Phylogenetic analyses were performed using the neighbor-joining method, based on the Kimura 2-parameter distance matrix and a transition-to-transversion ratio of 2.0, using MEGA software (Version 5.0) [25]. Tree topology was tested by bootstrap analysis with 1,000 replicates. HIV-1 recombinant analysis was carried out using Simpolt (Version 3.5.1; http://sray.med.som.jhmi.edu/SCRoftware/simplot/) [26].The parameters of bootscan analyses were as follows: window size of 350 bp, step size of 50 bp for near-full-length and half-genome sequences, window size of 200 bp, step size of 20 bp for *gag*, *pol*, and *vpr-env* gene fragments. The parameters of tree algorithm analysis were as follows: neighbor; distance model, Kimura; bootstrap replicate, 100; reference type, 50% consensus.

### Statistical analysis

Categorical variables are presented as number and percent, while continuous variables are presented as mean and standard deviation (SD). Some continuous variables were divided into categories to examine threshold effects. Fisher's exact probability and one-way ANOVA analyses were used to compare demographic and clinical characteristics of the study population. HIV-1 genotype distributions across study sites, ethnic groups, and diagnosis times were also compared using Fisher's exact probability. P-values below 0.05 were considered significant. Missing data received no special treatment and were simply excluded from analyses. All statistical analyses were performed using SPSS software (Version 17.0).

### Ethics statement

This study was reviewed and approved by the Medical Research Ethics Committee of No. 1 Hospital of China Medical University and written informed consent was obtained from all participants at the time blood samples were taken.

### Nucleotide sequence accession numbers

The sequences reported in this article are available in GenBank under accession numbers of JX070462–JX070532 and JX070534–JX070556 for *pol*, KC189061–KC189103 for VPR to *env*, KC189104–KC189153 for *gag* and KC898975–KC899015 for near full length or half genome sequences.

## Results

### Demographic and epidemiologic characteristics

The demographic and epidemiological characteristics of the 95 subjects included in the present study are shown in Table S1. The mean age of participants was 35±7.3 years. Most participants were male (93 of 95, 97.9%), of Dai ethnicity (45of 95, 47.4%), from Longchuan district (51of 95, 53.7%), and initially diagnosed with HIV-1 between 2000 and 2004 (36 of 95, 37.9%). Most blood samples were collected in 2009 (41of 95, 43.2%). Mean CD4^+^ T-cell count was 437±250 cells/µl and mean HIV-1 viral load was 4.47±0.68 log copies/ml.

### HIV-1 genotypes of IDUs in Dehong

**Figure 2:**
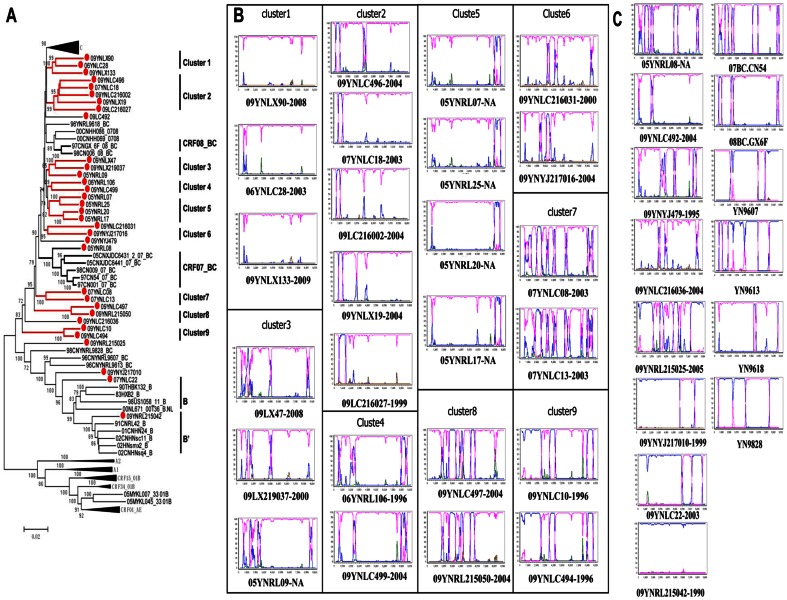
Phylogenetic tree and the boot-scanning plots of near-full-length sequences of HIV-1 strains isolated from Dehong. (A) The phylogenetic tree of 33 near-full-length sequences of from Dehong IDUs was constructed with MEGA 5.0 using the neighbor-joining method. The subtype reference sequences from the Los Alamos HIV Sequence Database were included as the reference sequences. The stability of the nodes was assessed by bootstrap analyses with1,000 replications. Only bootstrap values of more than 70 are shown at the corresponding nodes. The sequences from IDUs in Dehong are labeled by solid red circles. The nine clusters are shown in red lines. (B)The bootscanning plots of 25 near-full-length sequences in the nine clusters.(C)The bootscanning plots of 8 near-full-length sequences outside the clusters and 6 previously published near full-length sequences of B′/C recombinants in China. In the recombination analyses, a subtype C strain from India (95IN21068) ( pink) and a subtype B′ strain from Yunnan (RL42) (blue) were used as subtype references, and a CRF01_AE strain from Thailand (CM240) (green) was used as an out-group control. In (B) and (C), the number after the sequences ID showed the year of diagnosis of each case.

From the 95 HIV-positive IDUs in this study, 33(34.7%) near-full-length sequences, five (5.3%) 5′ half-genomes, three(3.2%) 3′ half-genomes, 88 (92.6%) *gag* gene sequences, 95 (100%) *pol* fragments, and 77 (81.1%) *vpr-env* fragments were successfully amplified and sequenced. Discrepancies in numbers of amplified fragments were due to the low volume of some specimens or low primer specificity. The genotype characterizations of HIV-1circulating among IDUs in Dehong were determined by phylogenetic and recombination analyses of the gene fragments of *gag*, *pol*, *vpr-env*, half-genome, and near-full-length sequences (Figure S1 and Figure 2). The genotype characterizations of each subject as well as the genotypes of their specific gene fragments are listed in Table 1. Due to the complexity of the recombinants present in this study, the genotypes may have been different depending on the gene region analyzed. For example, the genotype B′/C accounted for 13.0% (10 of 77) of sequences in the *vpr-env* region, but 63.6% (56 of 88) of sequences in the *gag* gene region. Because of discrepancies such as these, only the 91 samples with near-full-length or half-genome sequences or at least 2 sequences of *pol*, *gag* and *vpr-env* fragments were used in the following analyses on genotype-related factors.

**Table 1:** HIV-1 genotype characterizations of Dehong IDU subjects.

Sequence ID	Near-full-length or half genome	*gag* (790–2292)	*pol* (2253–3318)	*vpr-env* (5671–6449)	Genotype§
05YNLC186		C	B′C	C	B′C
05YNLC187*			B′		B′
05YNRL01	CB′C†	CB′C	B′C	C	B′C
05YNRL05	C01C01C†	C	01C		CRF01_AE/C
05YNRL07	CB′C	C	C	C	B′C
05YNRL08	CB′CB′CB′C	C B′C B′	B′C	CB′	B′C
05YNRL09	CB′CB′CB′C	C B′C B′	B′C	C	B′C
05YNRL17	CB′C	CB′C	C	C	B′C
05YNRL19		CB′C	B′C	C	B′C
05YNRL20	CB′C	CB′C	C	C	B′C
05YNRL25	CB′C	CB′C	C	C	B′C
05YNRL34		CB′CB′C	B′	B′C	B′C
05YNRL35		B′	B′	B′	B′
05YNRL43		C	CRF01_AE	C	CRF01_AE/C
06YNLC02		C	B′C	C	B′C
06YNLC04*			B′		B′
06YNLC05		B′	B′		B′
06YNLC07		CB′C	B′C		B′C
06YNLC15			B′C	CB′C	B′C
06YNLC16		CB′C	B′C	C	B′C
06YNLC17*			B′C		B′C
06YNLC19		CB′	B′C	C	B′C
06YNLC21		CB′C	B′C	C	B′C
06YNLC235			B′C	C	B′C
06YNLC26		CB′C	B′C		B′C
06YNLC27		CB′C	B′C	C	B′C
06YNLC28	C	CB′C	C	C	B′C
06YNLC30		CB′C	B′C	C	B′C
06YNLC31		CB′C	B′C	C	B′C
06YNLC32	C‡	CB′C	B′C	C	B′C
06YNLC38		CB′C	B′C	C	B′C
06YNLC40		B′C	B′C	C	B′C
06YNLC45		CB′C	B′C	C	B′C
06YNLC49		C	B′C	CB′C	B′C
06YNLC51		C B′	B′		B′C
06YNLC52		C	B′C	C	B′C
06YNLC53		CB′C	B′C	C	B′C
06YNLC54		CB′C	B′C	C	B′C
06YNLC55		CB′C	B′C	C	B′C
06YNRL106	CB′CB′CB′C	CB′CB′C	B′C	C	B′C
06YNRL115		C	C	C	C
07YNLC08	CB′CB′CB′CB′CB′CB′CB′C	CB′CB′C	B′C	CB′C	B′C
07YNLC13	CB′CB′CB′C†	CB′C	B′C		B′C
07YNLC18	CB′C	CB′C	B′C	C	B′C
07YNLC22	B′CB′C	B′	B′	B′	B′C
09YNLC10	B′CB′CB′C	B′	B′C	C	B′C
09YNLC216002	CB′C	CB′C	B′C	C	B′C
09YNLC216003		CB′CB′	B′C		B′C
09YNLC216004		CB′CB′	B′C	C	B′C
09YNLC216005	C‡	CB′C	B′C	C	B′C
09YNLC216017			C	B′	B′C
09YNLC216018		CB′C	B′C	C	B′C
09YNLC216027	CB′C	CB′C	B′C	C	B′C
09YNLC216029		B′	B′	C	B′C
09YNLC216031	CB′CB′CB′C	C	B′C	C	B′C
09YNLC216036	CB′CB′CB′C	C	B′	C	B′C
09YNLC216038		B′	B′C	B′	B′C
09YNLC216043		B′	B′	B′	B′
09YNLC216053		CB′C	B′C	C	B′C
09YNLC216055		CRF01_AE	CRF01_AE	CRF01_AE	CRF01_AE
09YNLC492	CB′CB′C	CB′C	B′C	C	B′C
09YNLC494	B′CB′CB′C	B′	B′C	C	B′C
09YNLC496	CB′CB′C	CB′C	B′C	C	B′C
09YNLC497	CB′CB′CB′C	C	B′C	CB′C	B′C
09YNLC499	CB′CB′CB′C	CB′C	B′C	C	B′C
09YNLX133	C	C	C	C	C
09YNLX19	CB′C	CB′C	C	C	B′C
09YNLX219003		CB′C	C	C	B′C
09YNLX219006		CB′C	01C	CRF01_AE	CRF01_AE/B/C
09YNLX219028		CB′C	C	C	B′C
09YNLX219037	CB′C	CB′CB′	B′C	C	B′C
09YNLX219050*			B′		B′
09YNLX219051		CB′C	C		B′C
09YNLX219053		CB′	B′		B′C
09YNLX227203	B′CB′C01CB′CB′†	CB′	B′C		CRF01_AE/B/C
09YNLX47	CB′CB′CB′C	CB′CB′	B′C	C	B′C
09YNLX90	C	C	C	C	C
09YNRL215005		CB′C	C		B′C
09YNRL215009	B′†	B′	B′	B′	B′
09YNRL215012		C	C	C	C
09YNRL215013		C	C	C	C
09YNRL215025	B′CB′CB′CB′	B′	B′C	CB′C	B′C
09YNRL215030		CB′C	B′C	C	B′C
09YNRL215042	B′	B′	B′	B′	B′
09YNRL215050	CB′CB′CB′C	C	B′C	CB′C	B′C
09YNRL215054		CB′	B′	C	B′C
09YNYJ217006		01B′C	B′C01		CRF01_AE/B/C
09YNYJ217010	B′CB′	B′	B′	B′	B′C
09YNYJ217013		CB′C	C	C	B′C
09YNYJ217016	CB′CB′C	CB′C	B′C	C	B′C
09YNYJ217036	CB′C‡	C	B′C	C	B′C
09YNYJ217040		C	C	CB′C	B′C
09YNYJ473	CB′CB′C†	CB′	B′C		B′C
09YNYJ479	CB′CB′CB′C	CB′C	C	CB′C	B′C
09YNYJ217055		CB′C	C		B′C

* These sequences were not included in the subtype related factors analysis.

† 5′ half genome sequences.

‡ 3′ half genome sequences.

§ Genotype was determined based on all sequences available.

### Factors associated with HIV-1 genotypes

Subjects were grouped into one of four categories (B′, B′/C, C, and Other) to represent their HIV-1 genotypes (Table 1).The genotype distribution of the 91 IDUs used for genotype-related factors analyses were as follows: 75 (82.4%) B′/C recombinants, 5 (5.5%) B′ genotypes, five (5.5%) C genotypes, and six (6.6%) other genotypes, including three that were B′/C/CRF01_AE, two that were C/CRF01_AE, and one that was pure CRF01_AE. The relationships of HIV-1 genotypes to the demographic and epidemiologic features of study population are summarized in Table 2. A statistically significant association was observed between HIV-1 genotype and age (p = 0.034), district (p = 0.022), and year of diagnosis (p = 0.004), suggesting both geographical and temporal differences in the distributions of the different HIV-1 genotypes. No other statistically significant associations were detected.

**Table 2:** Demographic and epidemiologic characteristics of the study population relative to their HIV-1 genotypes.

		HIV-1 Genotype				
Characteristics	Total (n = 91)	B′/C (n = 75)	B′ (n = 5)	C (n = 5)	Others* (n = 6)	P-value†
Gender, N (%)						
Male	89 (100)	73 (82.0)	5 (5.6)	5 (5.6)	6 (6.7)	0.933
Female	2 (100)	2 (100.0)	0	0	0	
Age (years)						
Mean ± SD	35±7.3	35±7.3	43±6.1	33±7.0	30±4.6	0.034
Ethnic Group, N (%)						
Achang	2(100)	2 (100)	0	0	0	0.661
Dai	44(100)	34 (77.3)	2 (4.5)	4 (9.1)	4 (9.1)	
Han	19(100)	17 (89.5)	0	1 (5.3)	1 (5.3)	
Jingpo	26(100)	22 (84.6)	3 (11.5)	0	1 (3.8)	
District, N (%)						
Longchuan	48 (100)	45 (93.8)	2 (4.2)	0	1 (2.1)	0.022
Luxi	10 (100)	7 (70.0)	0	2 (20.0)	1 (10.0)	
Ruili	23 (100)	15 (65.2)	3 (13.0)	3 (13.0)	2 (8.7)	
Yingjiang	10 (100)	8 (80.0)	0	0	2 (20.0)	
Diagnosis Year						
Before 1995	15 (100)	11 (73.3)	2 (15.4)	2 (13.3)	0	0.004
1996–1999	18 (100)	16 (88.9)	0	1 (5.6)	1 (5.6)	
2000–2004	35 (100)	33 (94.3)	2 (5.7)	0	0	
After 2005	9 (100)	4 (44.4)	0	2 (22.2)	3 (33.3)	
Unknown	14 (100)	11 (78.6)	1 (7.1)	0	2 (14.3)	
CD4^+^ T Cell Count (cells/µl)						
Mean ± SD	435±253	425±248	493±275	576±262	399±321	0.568
HIV-1 Viral Load (log copies/ml)						
Mean ± SD	4.49±0.68	4.47±0.70	4.79±0.56	4.87±0.72	4.33±0.52	0.524

SD: standard deviation.

* The “other” category is used to group subjects with more rare genotypes including B′/C/CRF01_AE and C/CRF01_AE recombinants and pure CRF01_AE.

† Statistical significance was determined by Fisher's exact test and one-way ANOVA analysis.

### Geographical distribution of HIV-1 genotypes

As shown in Table 2 and Figure 1B, the prevalence of B′/C recombinants was more than 70% in Longchuan, Luxi, and Yingjiang, with the highest prevalence observed in Longchuan (45 of 48, 93.8%),where the prevalence of subtype C was found to be the lowest (0 of 48) among the four districts. By contrast, the lowest prevalence of B′/C recombinants was found in Ruili (15 of 23, 65.2%), where pure subtype B′ and subtype C was at its highest prevalence (6 of 23, 26.0%). This higher prevalence of pure subtype B′ and subtype C in Ruili relative to other districts was statistically significant (p = 0.022). It is important to note that 5 of the 6 pure subtype B′ or subtype C genotype HIV-positive subjects were diagnosed before 1995 and were between 40 and 48 years old.

**Figure 3:**
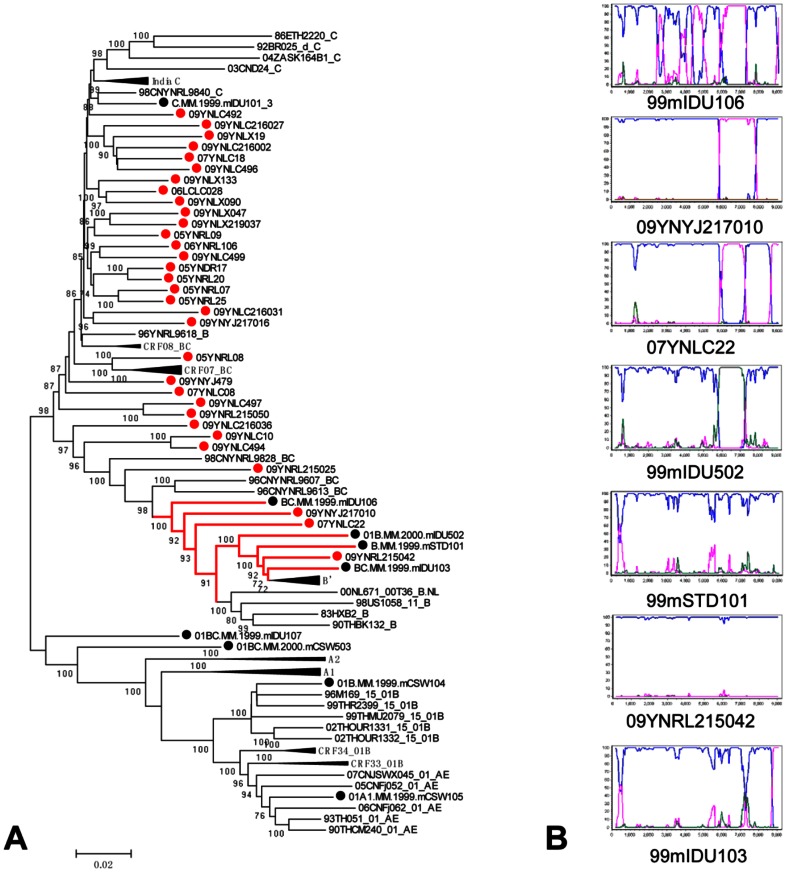
The phylogenetic tree and the bootscanning plots of near-full-length sequences of HIV-1 strains isolated from Dehong and Myanmar. (A) The phylogenetic tree of near-full-length sequences from IDUs in Dehong (red circles) and Myanmar (black circles).(B)The bootscanning plots of near-full-length sequences from the cluster that included sequences from IDUs both in Dehong and in Myanmar with bootstrap values above 70. In the recombination analyses, a subtype C strain from India (95IN21068) (pink) and a subtype B′ strain from Yunnan (RL42) (blue) were used as subtype references, and a CRF01_AE strain from Thailand (CM240) (green) was used as an out-group control.

In order to examine potential geographical linkage with nearby regions, we compared the HIV-1 genotypes of IDUs in Dehong with data from previous HIV-1 genotyping studies on IDUs in Myanmar. As shown in Table 3, significantly more B′/C recombinants and fewer other genotypes were found in the Dehong study population when compared with findings from both northern and central Myanmar (p<0.001) [20], [27]. In order to elucidate the phylogenetic relationships between HIV-1 strains among IDUs in Dehong and Myanmar, neighbor-joining tree analysis was conducted based on near-full-length sequences and gene fragments of *gag*, *pol*, *vpr-env* regions from this study as well as two previous studies (Figure 3 and Figure S2). Although various HIV-1 recombinant forms, especially B′/C recombinants, were detected in both Dehong and Myanmar, none of the recombinant sequences from Dehong and Myanmar clustered together with a bootstrap value above 70 in near-full-length neighbor-joining tree analysis except for a cluster that included three sequences from Dehong IDUs, three sequences from Myanmar IDUs, and one sequence from a Myanmar patient who acquired HIV via sexual contact (bootstrap value 100; Figure 3A). However, breakpoint analysis demonstrated these sequences had different recombination patterns or no breakpoints (Figure 3B).Two other clusters of Dehong and Myanmar sequences were observed in *pol* neighbor-joining tree analysis (bootstrap values of 92 and 98). However, these sequences did not cluster together in the *gag* and *vpr-env* neighbor-joining tree analyses (Figure S2), suggesting that although the recombinants from Dehong and Myanmar are closely related, the recombination events that gave rise to these strains occurred independently in Dehong and Myanmar.

**Table 3:** Comparison of HIV-1 genotype distributions among IDUs in Dehong and Myanmar.

	Year of sampling	Region of sampling	Number of samples	Genome regions analyzed	Genotype				P-value†
					B′/C	B′	C	Other*	
Present Study	2005–2009	Dehong	91	*pol*	75 (82.4)	5 (5.5)	5(5.5)	6 (6.6)	-
Pang *etal. * [*20*]	2009	Northern Myanmar	79	*gag* p17, *pol*, *vif-env* C2V3	37(46.8)	3(3.8)	6(7.6)	33(41.8)	<0.001
Takebe.*etal. * [*27*]	1999–2000	Central Myanmar	21	*gag* p17,*env*C2/V3	2(9.5)	7(33.3)	4(19.0)	8(38.1)	<0.001
Qiu. *etal. * [*15*]	1996–1998	Dehong	54	*env* C2V3,near-full-length sequences	4(7.4)	49 (90.7)	1(1.9)	0	<0.001
Yang. *etal. * [*11*]	2000–2001	Dehong	14	*gag*-RT*env* C2V3	9(64.3)	4 (28.6)	0	1 (7.1)	0.032
Zhang. *et al. * [*16*]	2003	Dehong	13	*gag* p17 and RT	8(61.5)	2 (15.4)	0	3 (23.1)	0.087

* The “other” category is used to group subjects with more rare genotypes including B′/C/CRF01_AE and C/CRF01_AE recombinants and pure CRF01_AE.

† Statistical significance of the distribution of genotypes between previous study and present study was determined by Fisher's exact test.

### Temporal distribution of HIV-1genotypes

In our samples, we observed a trend of increasing prevalence of HIV-1 B′/C recombinants in IDUs from Dehong who were diagnosed at different periods (Table 2). In those diagnosed before 1995, 73.3% carried B′/C recombinant genotypes, whereas in those diagnosed between 1996 and 1999, the proportion had increased to 88.9%, and then between 2000 and 2004, had increased further to 94.3%. However, after 2005, the prevalence of B′/C recombinants dropped dramatically to 44.4%. Over this same period, from prior to 1995 to after 2005, a few pure subtype B′ and pure subtype C genotypes were detected, though these were very rare.

In order to more closely examine temporal changes in genotype distribution, we compared our results with data from previous HIV-1 genotyping studies among Dehong IDUs. As summarized in Table 3, HIV-1 subtype B′ was the predominant form (49 of 54, 90.7%) in samples collected from 1996 to 1998 [15], while B′/C recombinants were less common (4 of 54, 7.4%). In contrast, studies performed on samples collected from 2000 to 2001 [11] and in 2003 [16] document the prevalence of B′/C recombinants as being greater than 60% and the prevalence of subtype B′ at less than 30%. Taken together, our results and those of previous studies suggest that while B′/C recombinant forms are arising continuously and have become the dominant HIV-1 subtype in Dehong prefecture, pure subtype B′ and pure subtype C genotypes have not yet disappeared from this population.

### Evolution of HIV-1 recombinants in Dehong

**Figure 4:**
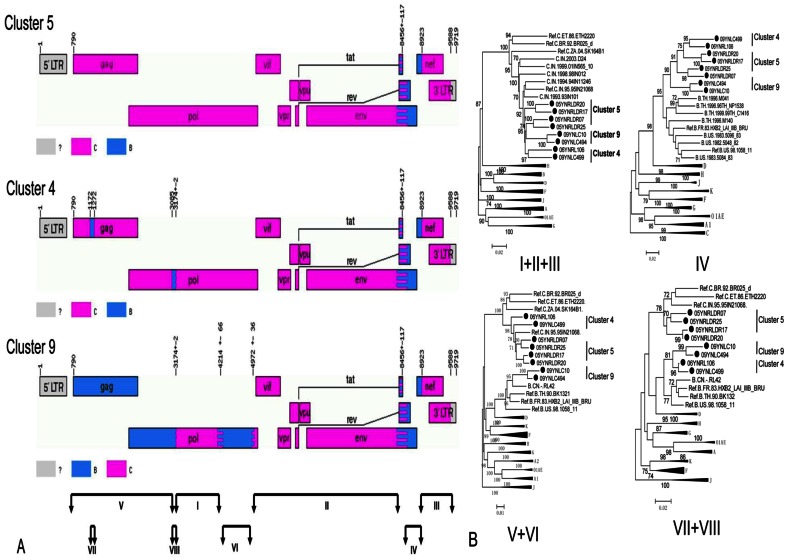
Recombination map of sequences in clusters 4, 5, and 9, and sub-region neighbor-joining tree. (A)The recombination map of sequences in the clusters 4, 5, and 9 of the near-full-length sequences phylogenetic tree.(B)The sub-region tree of corresponding regions as shown by roman numerals. The closely related sequences from Asian countries and subtype reference sequences are included.

**Figure 5:**
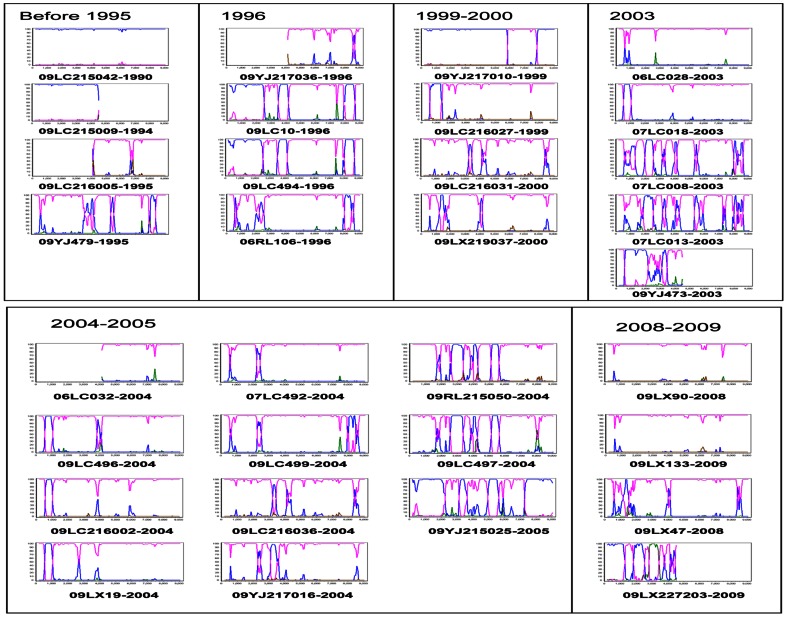
The bootscanning plots of 26 near-full-length sequences and 6 half-genome sequences of HIV-1 strains isolated from Dehong IDUs with diagnosis year in chronological order. In the recombination analyses, a subtype C strain from India (95IN21068) (pink) and a subtype B′ strain from Yunnan (RL42) (blue) was used as subtype references, and a CRF01_AE strain from Thailand (CM240) (green) was used as an out-group control.

To further explore the evolutionary relationships of B′/C recombinants in Dehong, and to investigate the possibility of second generation recombination, we compared the recombination patterns of 33 near-full-length sequences from IDUs diagnosed at different time periods. In the phylogenetic tree generated using near-full-length sequences, 25sequences were clearly grouped into nine clusters (bootstrap value >80; Figure 2A). In addition, seven B′/C strains and one pure B′ strain was observed. The sizes of the nine clusters ranged from two to five sequences. The results of our recombination analysis reinforced the findings of our phylogenetic analysis: at least 16 different recombination patterns were observed, none of which match those previous reported in Dehong (Figure 2B and Figure 2C). In general, while the 25 sequences in clusters have recombinants from B′ and C subtypes, it is difficult to resolve the evolutionary relationships between them. However, it is interesting to note that the 8 sequences in clusters 4, 5, and 9 all had a B′ fragment in the *env-nef* region and all shared the same breakpoints (Figure 2B and Figure 4A). The sub-region tree suggested that sequences in clusters 4, 5, and 9 had a common subtype C backbone and B′ fragment insertion in the *env-nef* region (Figure 4B). However, the sequences in clusters 4 and 9 contained more B′ fragment insertions in the 5′ half-genome, compared with those in cluster 5, implying the possibility of second generation recombination. In addition, the B′ fragments of clusters 4 and 9 in 5′ half-genome clustered together, implying a homologous relationship (Figure 4B).The recombination maps of the 26 near-full-length sequences and 6 half-genomes from patients that have the diagnoses time are presented in Figure 5. These data show that more pure B′ and pure C sequences are observed in earlier groups, while more B′/C recombinant forms are observed in later groups. However, in the four sequences from subjects diagnosed between 2008 and 2009, two pure subtype C, one B′/C recombinant, and one CRF_01_AE/B′/C recombinant were observed.

## Discussion

In the present retrospective study, using blood samples collected from 95 HIV-positive IDUs from Dehong between 2005 and 2009, we determined HIV-1 *gag* gene, partial *pol* gene, *vpr-env* region, half-genome, and near-full-length nucleotide sequences and analyzed their phylogenetic relationships and recombinant structures. These were compared to the demographic and epidemiological data of our study population as well as to results from prior HIV-1 molecular epidemiology studies both in Dehong and in Myanmar.

The main finding of our study was the significant predominance of HIV-1 B′/C recombinant forms among Dehong IDUs (Table 1 and Figure 1B), which have structures that have not previously been documented in the literature (Figure 2). The different B′/C recombinant forms we have found may have been generated via recombination events occurring not only between subtype B′ and subtype C forms, but also between different B′/C forms via second generation recombination events. The phylogenetic and recombination analysis on the near-full-length sequences supported the idea that sequences in clusters 4 and 9 may be second generation recombinant forms that have arisen from B′ and B′/C recombinants in cluster 5 (Figure 2). We believe this may be further supported when the year of diagnosis is considered. Three of the four subjects each in clusters 4 and 9 were diagnosed in 1996 (the fourth was diagnosed in 2004). Unfortunately, diagnosis year data was unavailable for the four sequences in cluster 5, so we cannot draw conclusions about these potential second generation recombinants based on subject diagnosis year. However, our phylogenetic analyses showed likely evolutionary relationships among some B′/C recombinants. Furthermore, the increasing complexity of recombination patterns suggests that there is a high frequency of HIV-1 recombination events within this population and new recombinant forms are arising continuously (Figure 5). This phenomenon has been documented in neighboring Myanmar, where 86.1% of samples in one study were found to be recombinants [27]. The co-circulation of multiple lineages of HIV-1 strains in pre-existing transmission networks with high-risk behaviors (like that of IDUs) could easily fuel the generation of a variety of new recombinant strains in China.

This idea is further supported by evaluating our findings in the context of previous molecular epidemiological studies of HIV-1 recombinant forms in Dehong prefecture. The high prevalence of B′/C recombinants in our sample is consistent with previous observations by Yang *et al.* for samples collected between 2000 and 2001 [11], and Zhang *et al.* for samples collected in 2003 [16], but is different from samples collected by Qiu *et al.* between 1996 and 1998 [11], [15], [16], in which HIV-1 B′ subtype virus was most common. While this makes sense in light of the fact that B′/C recombinant forms first became common in Dehong in 1996, shortly after the pure HIV-1 subtype C virus was introduced [11]–[16], [18]. These data taken together, point to an increase in the prevalence of B′/C recombinants over time (Table 3).

Surprisingly however, two of the four cases diagnosed between 2008 and 2009 (with seronegative records within six months), were identified as having pure subtype C strains, seemingly contrary to the trend of increasing B′/C recombinant prevalence. It is important to note that in recent years, many HIV prevention policies have been implemented by the Chinese government. In Dehong, needle exchange programs began in 2003, and the first methadone maintenance treatment clinics were established in 2005. These newly-infected subjects carrying pure subtype C viral strains are therefore not infected with multiple strains of HIV-1.This may be an indication that the HIV prevention policies have achieved some success, even in areas with high rates of recombination such as Dehong.

Our results regarding the geographical distribution of HIV-1 genotypes are notable and in line with our analysis of the HIV-1 genotype temporal distribution in Dehong (Table 2and Figure 5). While the prevalence of HIV-1 B′/C recombinants is relatively high in all four districts, significantly higher prevalence of pure subtypes B′ and C were observed in Ruili, relative to other regions. A possible explanation for this finding is that subjects from Ruili were infected with HIV in the early- and mid-1990s when fewer B′/C strains were present in the emerging epidemic in Dehong (5 of 23, 21.7% of Ruili subjects were diagnosed before 1995, data not shown).

Another significant finding is the relationship of the HIV epidemics in Dehong and neighboring Myanmar. Dehong is the most active channel for the trafficking of illicit drugs from Myanmar and other golden triangle countries into China. A high prevalence of diverse forms of HIV-1 B′, C and CRF01_AEintersubtyperecombinantshas been reported in Myanmar [27], [28]. With the most recent report from northern Myanmar finding that 86.1% of HIV-positive IDUs carry intersubtype recombinant forms of the virus [20]. The recombinant forms identified in this report include all four possible strains formed from recombination events between pure subtype B′, pure subtype C, and CRF01_AE. In addition, most recombinants have distinguishable chimeric patterns, forming 64 URFs that are dissimilar to previously identified CRFs and other URFs in Asia. In the present study, we detected a similar prevalence of intersubtype recombinant forms in Dehong (80 of 91, 87.9%), which prompted us to consider the possibility of a close relationship between the HIV epidemics in the two regions. However, comparison of the phylogenetic relationship among recombinants in Dehong and Myanmar revealed that none of the available sequences shared the same recombination patterns. Taken together, these data suggest that Dehong and Myanmar are both experiencing high frequency and complex HIV-1 intersubtype recombination events, but that they are largely independently of each other.

Our study had several limitations. Firstly, comparison of results from this study to previous molecular epidemiological observations is difficult due to inter-study variability of the gene regions analyzed. For example, Qiu *et al.* used the C2V3 region of *env* gene to determine genotype [15]. Because breakpoints are more commonly documented in the *pol* gene [8]–[11], it could be that the prevalence rates of HIV-1 B′/C recombinant genotypes in the Qiu *et al.* study were underestimated. Secondly, because subjects who were diagnosed in earlier years could have had a greater probability of being infected with HIV multiple times (compared to subjects diagnosed in later years), their samples may have presented with more recombinants. Thirdly, our dataset, while much larger in sample size than previous studies, did have some missing values. This could have resulted in the under- or overestimation of the association of HIV-1 B′/C recombinant genotypes with demographic or epidemiologic factors.

In conclusion, our study documents a high and increasing prevalence of HIV-1 B′/C recombinants among Dehong IDUs since 1996, shortly after the subtype C virus was introduced in the region. Further, the increasing predominance of multiple, new, forms of unique B′/C recombinant strains suggests that HIV-1 intersubtype recombination is extensive and ongoing among Dehong IDUs. These findings highlight the urgency of strengthening monitoring efforts and implementing effective measures to reduce transmission in this population. It also underscores the need for further research on the rate of spread of these forms outside of Dehong and the potentially altered biology of these new recombinant forms. The increasing mobility of people across local and international borders, presents a high potential for the rapid evolution of an HIV strain with exaggerated transmission efficiency, virulence, or drug resistance, which is a global problem of critical importance.

## Supporting Information

Figure S1 **Neighbor-joining trees of gag, pol and vpr to env fragments from Dehong IDUs.** Neighbor-joining trees were built based on the sequences of 88 gag gene (A), 95 pol gene (B) and 77 vpr to env gene fragments. The subtype references sequences from the Los Alamos HIV Sequence Database were included as the reference sequences. The stability of the nodes was assessed by bootstrap analyses with 1000 replications. Only bootstrap values of more than 70 are shown at the corresponding nodes. The sequences from IDUs in Dehong are labeled by red solid circles, and previous published sequences from Dehong are labeled by black triangles. The clusters that including sequences from Dehong both in this study and previous published sequences were showed in red line.
(TIF)

Figure S2 **Neighbor-joining trees of gag p17, pol and vpr to env fragments from IDUs in Dehong and Myanmar.** Neighbor-joining trees were built based on the gag P17 sequences of 88 IDUS in this study and 75 IDUs in Myanmar (A), pol gene from 95 IDUs in this study and 69 IDUs in Myanmar (B) and vpr to env gene fragments from 77 IDUs in this study and 64 IDUs in Myanmar (C). The subtype references sequences from the Los Alamos HIV Sequence Database were included as the reference sequences. The stability of the nodes was assessed by bootstrap analyses with 1000 replications. Only bootstrap values of more than 70 are shown at the corresponding nodes. The sequences from IDUs in Dehong are labeled by red solid circles, and previous published sequences from Myanmar are labeled by black solid circles. The clusters that including sequences from both Dehong and Myanmar were showed in red line.
(TIF)

Table S1 **Demographic and clinical characteristics of the study population.**
(DOC)

## References

[1] Ma Y, Li Z, Zhang K, Yang W, Ren X, et al (1990) [HIV infections detected in drug users for the first time in our country]. Chinese Journal of Epidemiology 11: 184–185.

[2] Zhang J, Cheng H, Zhano S, Yang W, Ma Y, et al (1991) [Epidemiological survey on HIV infected cases in Ruili, Yunnan]. Chinese Journal of Epidemiology 12: 9.1878966

[3] Zheng X, Zhu L, G Y, Zhang G (1989) [China AIDS monitoring reports(1985–1989)]. Chinese Journal of Epidemiology 10: 65.2736616

[4] Y S, Zeng Y, Chen Z, Zhao S, Ma Y, et al (1991) [Isolation of viruses from HIV infected individuals in Yunnan]. Chinese Journal of Epidemiology 12: 129–135.1713810

[5] X Z, C T, Yang G, Xia M, Zhu L, et al (1991) [A preliminary study on the behavior of 225 drug abusers and the risk factors of HIV infection in Ruili county Yunnan Province]. Zhonghua Liu Xing Bing Xue Za Zhi 12: 12–14. 1878954

[6] Zheng XW, Zhang JP, Tian CQ, Cheng HH, Yang XZ, et al (1993) Cohort study of HIV infection among drug users in Ruili, Longchuan and Luxi of Yunnan Province, China. Biomed Environ Sci 6: 348–351. 8198751

[7] Yu ES, Xie Q, Zhang K, Lu P, Chan LL (1996) HIV infection and AIDS in China, 1985 through 1994. Am J Public Health 86: 1116–1122. 8712271 10.2105/ajph.86.8_pt_1.1116 PMC1380623

[8] Piyasirisilp S, McCutchan FE, Carr JK, Sanders-Buell E, Liu W, et al (2000) A recent outbreak of human immunodeficiency virus type 1 infection in southern China was initiated by two highly homogeneous, geographically separated strains, circulating recombinant form AE and a novel BC recombinant. J Virol 74: 11286–11295. 11070028 10.1128/jvi.74.23.11286-11295.2000 PMC113233

[9] Shao Y, Zhao F, Yang W, Zhang Y, Gong X (1999) [The identification of recombinant HIV-1 strains in IDUs in southwest and northwest China]. Zhonghua Shi Yan He Lin Chuang Bing Du Xue Za Zhi 13: 109–112. 12569772

[10] Su L, Graf M, Zhang Y, von Briesen H, Xing H, et al (2000) Characterization of a virtually full-length human immunodeficiency virus type 1 genome of a prevalent intersubtype (C/B′) recombinant strain in China. J Virol 74: 11367–11376. 11070037 10.1128/jvi.74.23.11367-11376.2000 PMC113242

[11] Yang RG, Xia XS, Kusagawa S, Zhang CY, Ben KL, et al (2002) On-going generation of multiple forms of HIV-1 intersubtype recombinants in the Yunnan Province of China. AIDS 16: 1401–1407. 12131217 10.1097/00002030-200207050-00012

[12] Shao Y, Zhao Q, Wang B, Chen Z, Su L, et al (1994) [Sequence analysis of HIV env genes among HIV infected drug injecting users In Dehong epidemic aera of Yunnan province,China]. Chinese Jouranl of virology 10: 291–299.

[13] Shao Y, Y G, Q Z, Y Z, Zhang J, et al (1995) [Genetic variations and molecular epidemiology of the Ruili HIV-1 strains of Yunnan in 1995]. Chinese Jouranl of virology 12: 10–17.

[14] Guan Y, Chen J, Shao Y, Zhao Q, Zeng Y (1997) Subtype and sequence analysis of the C2- V3 region of gp120 genes among human immunodeficiency virus infected IDUs in Ruili epidemic area of Yunnan Province of China. Chinese J Exp Clin Virol 11: 8–13. 15619893

[15] Qiu ZZ, Xing H, Wei M, Duan YJ, Zhao QB, et al (2005) Characterization of five nearly full-length genomes of early HIV type 1 strains in Ruili City: Implications for the genesis of CRF07_BC and CRF08_BC circulating in China. Aids Research and Human Retroviruses 21: 1051–1056. 16379609 10.1089/aid.2005.21.1051

[16] Zhang Y, Lu L, Ba L, Liu L, Yang L, et al (2006) Dominance of HIV-1 subtype CRF01_AE in sexually acquired cases leads to a new epidemic in Yunnan province of China. PLoS Med 3: e443. 17105339 10.1371/journal.pmed.0030443 PMC1635743

[17] Wang B, Lu X, Shao J, Shao Y, Zeng Y (1998) [Comparison of Amino Acid Consensus sequences of Membrane Protein V3 Region between International and Local Epidemic HIV_1 Strains from Yunnan During 1992–1994]. VIROLOGICA SINICA 13: 226–230.

[18] Li DQ, Zheng XW, Zhang GY (1996) Study on the distribution HIV-1 C subtype in Ruili and other counties, Yunnan, China. Zhonghua Liu Xing Bing Xue Za Zhi 17: 337–339. 9387597

[19] Liu J, Zhang C (2011) Phylogeographic analyses reveal a crucial role of Xinjiang in HIV-1 CRF07_BC and HCV 3a transmissions in Asia. PLoS One 6: e23347. 21858079 10.1371/journal.pone.0023347 PMC3155551

[20] Pang W, Zhang CY, Duo L, Zhou YH, Yao ZH, et al (2012) Extensive and complex HIV-1 recombination between B ′, C and CRF01_AE among IDUs in south-east Asia. AIDS 26: 1121–1129. 22333750 10.1097/QAD.0b013e3283522c97

[21] Han X, Zhang M, Dai D, Wang Y, Zhang Z, et al (2007) Genotypic resistance mutations to antiretroviral drugs in treatment-naive HIV/AIDS patients living in Liaoning Province, China: baseline prevalence and subtype-specific difference. AIDS Res Hum Retroviruses 23: 357–364. 17411368 10.1089/aid.2006.0094

[22] Zhang M, Han XX, Cui WG, Jia MH, Meng XD, et al (2008) The impacts of current antiretroviral therapy regimens on Chinese AIDS patients and their implications for HIV-1 drug resistance mutation. Jpn J Infect Dis 61: 361–365. 18806342

[23] Zhao B, Han X, Dai D, Liu J, Ding H, et al (2011) New Trends of Primary Drug Resistance Among HIV Type 1-Infected Men Who Have Sex with Men in Liaoning Province, China. AIDS Res Hum Retroviruses 10.1089/AID.2010.0119 21417755

[24] Salazar-Gonzalez JF, Salazar MG, Keele BF, Learn GH, Giorgi EE, et al (2009) Genetic identity, biological phenotype, and evolutionary pathways of transmitted/founder viruses in acute and early HIV-1 infection. J Exp Med 206: 1273–1289. 19487424 10.1084/jem.20090378 PMC2715054

[25] Tamura K, Dudley J, Nei M, Kumar S (2007) MEGA4: Molecular Evolutionary Genetics Analysis (MEGA) software version 4.0. Mol Biol Evol 24: 1596–1599. 17488738 10.1093/molbev/msm092

[26] Lole KS, Bollinger RC, Paranjape RS, Gadkari D, Kulkarni SS, et al (1999) Full-length human immunodeficiency virus type 1 genomes from subtype C-infected seroconverters in India, with evidence of intersubtype recombination. J Virol 73: 152–160. 9847317 10.1128/jvi.73.1.152-160.1999 PMC103818

[27] Takebe Y, Motomura K, Tatsumi M, Lwin HH, Zaw M, et al (2003) High prevalence of diverse forms of HIV-1 intersubtype recombinants in Central Myanmar: geographical hot spot of extensive recombination. AIDS 17: 2077–2087. 14502011 10.1097/00002030-200309260-00009

[28] Motomura K, Kusagawa S, Kato K, Nohtomi K, Lwin HH, et al (2000) Emergence of new forms of human immunodeficiency virus type 1 intersubtype recombinants in central Myanmar. AIDS Res Hum Retroviruses 16: 1831–1843. 11118069 10.1089/08892220050195793

