# Supplementary figures and images for: High Prevalence of HIV-1 Intersubtype B′/C Recombinants among Injecting Drug Users in Dehong, China
Source: PLoS One. 2013 May 31;8(5):e65337. doi: 10.1371/journal.pone.0065337 (PMC3669332; doi:10.1371/journal.pone.0065337)

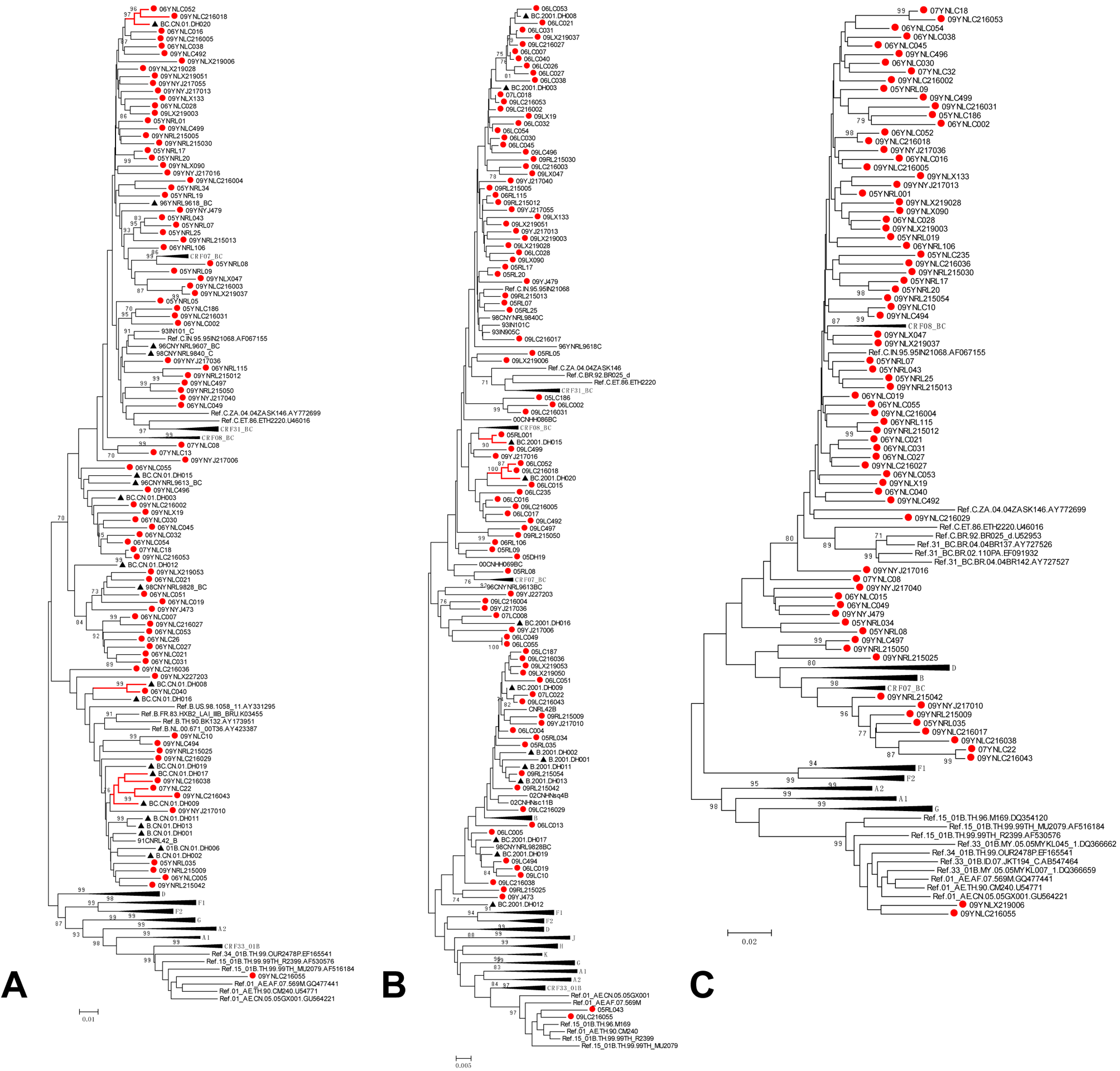

Supplement: Figure S1 — Neighbor-joining trees of gag, pol and vpr to env fragments from Dehong IDUs. Neighbor-joining trees were built based on the sequences of 88 gag gene (A), 95 pol gene (B) and 77 vpr to env gene fragments. The subtype references sequences from the Los Alamos HIV Sequence Database were included as the reference sequences. The stability of the nodes was assessed by bootstrap analyses with 1000 replications. Only bootstrap values of more than 70 are shown at the corresponding nodes. The sequences from IDUs in Dehong are labeled by red solid circles, and previous published sequences from Dehong are labeled by black triangles. The clusters that including sequences from Dehong both in this study and previous published sequences were showed in red line. (TIF) [file pone.0065337.s001.tif]

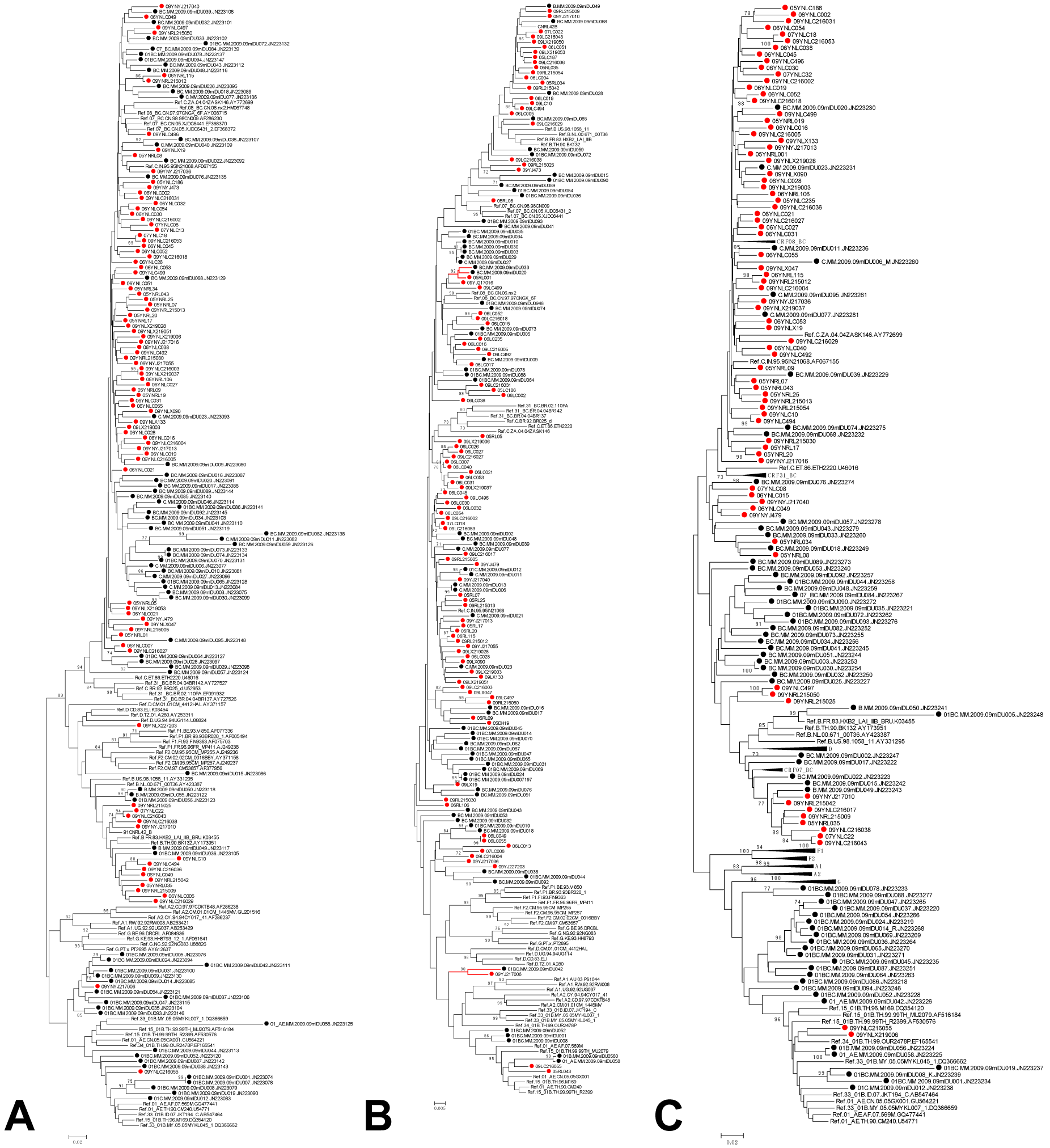

Supplement: Figure S2 — Neighbor-joining trees of gag p17, pol and vpr to env fragments from IDUs in Dehong and Myanmar. Neighbor-joining trees were built based on the gag P17 sequences of 88 IDUS in this study and 75 IDUs in Myanmar (A), pol gene from 95 IDUs in this study and 69 IDUs in Myanmar (B) and vpr to env gene fragments from 77 IDUs in this study and 64 IDUs in Myanmar (C). The subtype references sequences from the Los Alamos HIV Sequence Database were included as the reference sequences. The stability of the nodes was assessed by bootstrap analyses with 1000 replications. Only bootstrap values of more than 70 are shown at the corresponding nodes. The sequences from IDUs in Dehong are labeled by red solid circles, and previous published sequences from Myanmar are labeled by black solid circles. The clusters that including sequences from both Dehong and Myanmar were showed in red line. (TIF) [file pone.0065337.s002.tif]
