# Supplementary material for: High Prevalence of HIV-1 Intersubtype B′/C Recombinants among Injecting Drug Users in Dehong, China
Source: PLoS One. 2013 May 31;8(5):e65337. doi: 10.1371/journal.pone.0065337 (PMC3669332; doi:10.1371/journal.pone.0065337)
Supplement: Table S1 — Demographic and clinical characteristics of the study population. (DOC) [file pone.0065337.s003.doc]

**Table S1.** Demographic and clinical characteristics of the study population.

| Characteristics | All Cases  N (%) | Complete Cases^†^  N (%) |
| --- | --- | --- |
| Gender |  |  |
| Male | 93 (97.9) | 89 (97.8) |
| Female | 2 (2.1) | 2 (2.2) |
| Age |  |  |
| <30 | 22 (23.2) | 22 (24.2) |
| 30-40 | 48 (50.5) | 45 (49.5) |
| 40-50 | 22 (23.2) | 21 (23.1) |
| >50 | 3 (3.2) | 3 (3.3) |
| Mean ± SD^*^ | 35±7.2 | 35±7.3 |
| Nationality |  |  |
| Achang | 2 (2.1) | 2 (2.2) |
| Dai | 45 (47.4) | 44 (48.4) |
| Deang | 1 (1.1) | 0 |
| Han | 19 (20.0) | 19 (20.9) |
| Jingpo | 28 (29.5) | 26 (28.6) |
| Diagnosis Year |  |  |
| Before 1995 | 15 (15.8) | 15 (16.5) |
| 1996-1999 | 20 (21.1) | 18(19.8) |
| 2000-2004 | 36 (37.9) | 35 (38.5) |
| After 2005 | 9 (9.5) | 9 (9.9) |
| Unknown | 15 (15.8) | 14 (15.4) |
| Sampling Year |  |  |
| 2005 | 14 (14.7) | 13 (14.3) |
| 2006 | 28 (29.5) | 26 (28.6) |
| 2007 | 10 (10.5) | 10 (11.0) |
| 2008 | 2 (2.1) | 2 (2.2) |
| 2009 | 41 (43.2) | 40 (44.0) |
| Sampling Site |  |  |
| Longchuan | 51 (53.7) | 48 (52.7) |
| Luxi | 11 (11.6) | 10 (11.0) |
| Ruili | 23 (24.2) | 23 (25.3) |
| Yingjiang | 10 (10.5) | 10 (11.0) |
| CD4^+^ T Cell Count (cells/µl) |  |  |
| >500 | 34 (35.8) | 32 (35.2) |
| 200-500 | 48 (50.5) | 46 (50.5) |
| <200 | 13 (13.7) | 13 (14.3) |
| Mean ± SD^*^ | 436±250 | 435±253 |
| Viral Load (log copies/ml) |  |  |
| >5 | 20 (21.1) | 20 (22.0) |
| 4-5 | 51 (53.7) | 48 (52.7) |
| <4 | 20 (21.1) | 19 (20.9) |
| NA^‡^ | 4 (4.2) | 4 (4.4) |
| Mean ± SD^*^ | 4.47±0.68 | 4.49±0.68 |
| Overall | 95 (100.0) | 91 (100.0) |

^*^Standard deviation.

^†^ Due to the complexity of the recombinants present in this study, genotypes may have been different depending on the gene region analyzed. Therefore, only the 91 samples with near-full-length or half-genome sequences or at least 2 fragments of pol, gag and vpr-env sequences available were used in the following analyses.

^‡^ unavailable.
